# Medical Etiquette Tested by Practice

**Published:** 1914-06-20

**Authors:** 


### THE SCHOOL DOCTOR AND THE "G. P."

### Medical Etiquette Tested by Practice.

Nowadays, when whole-time school medical officers have been appointed by nearly all local authorities, it is highly desirable that all those who hold such positions should combine, in their own interests as well as in those of the profession generally. The Medical Officers of Schools Association is a useful body to join. It issues its own quarterly journal, *School Hygiene*, which deals principally with questions of outstanding interest to school doctors. It has, moreover, a strong and fully representative council, whose active interest in, and attention to, all matters pertaining to the work which the school doctor is called upon to perform have been proved in the past four years during which the Association has been at work.

The position of the school doctor in relation to the general practitioner should not, ordinarily, give rise to any difficulties. Where it does so, want of tact or of that fine feeling of brotherly charity and goodwill which is the basis of that popularly misunderstood intangibility, medical etiquette, is usually the underlying cause. The school doctor is an adviser, appointed by a local authority to investigate the hygiene of a section of the population. He is not responsible for treatment: in many cases he is definitely debarred, under the terms of his agreement, from carrying out, or even suggesting, lines of treatment for individual cases. As an adviser, he can only report and note defects which come under his observation. Where parents are present at the routine inspections, it is his duty to draw their attention to defects discovered by him in the children: he must, if he is to do any good, suggest that these defects should be treated. That is obvious. But there is some difference in the various ways in which his suggestions may be conveyed to the parent. Where they have a medical adviser, a family doctor, he should tactfully suggest that the child may, in the first instance, be taken to the family doctor for the latter's examination and report. Where no family doctor is available, he should suggest a reference to the nearest practitioner, if the parents' circumstances are such that they can afford private treatment: where they cannot afford such treatment, the treatment centre and the children's hospital supply the deficiency. In theory, this line of policy is easy enough. But in practice conditions sometimes make it difficult.

The school doctor may discover that the child suffers from a mild degree of astigmatism which, in his opinion, needs correction by suitable glasses. The parent reports that the family practitioner "does not think the child needs glasses." Here there is a direct conflict of opinion, in which the services of a disinterested third party are desirable. Similar conflict of opinion often occurs between the school doctor and the hospital out-patient officer, especially in cases in which the former has advised operation for septic tonsils or chronic appendicitis, and is much more difficult to settle satisfactorily. It may be thought that on matters of in-patient treatment, especially the necessity for operation in cases of chronic appendicitis, the hospital surgeon speaks with far greater weight and authority than does the school doctor. As a matter of fact that is not always the case. The school doctor has probably had the child under observation for several months: he has carefully weighed every consideration in the case, and he has generally had considerable experience in regard to children's diseases. The verdict of the hospital consultant (often an inexperienced young resident, who takes out-patients for a senior absent on holiday) is given after one examination, which may or may not have been thorough. The writer recalls two recent cases in which the school doctor advised operation in children suffering from chronic catarrhal appendicitis, in both of which hospital surgeons refused operation, with disastrous results several months later. The truth is that many hospital surgeons still imagine that chronic catarrhal appendicitis in children, for its adequate clinical demonstration, needs the presence of a train of symptoms that are due to subacute peritonitis, and refuse to recognise the gravity of so-called slight cases, which in children may suddenly develop seriously.

In such circumstances the school doctor can only stick to his guns and counsel parents to take the advice of another hospital surgeon. His own knowledge of certain members of the staff of particular hospitals will enable him to suggest to which institution the child should be taken. Here there can be no question of patronage or advertising: all that is done is to supply the parents with particular information, which in the circumstances it is justifiable and legitimate to supply. A recommendation to a private practitioner would, in the circumstances, be unethical; but it is no more unethical for the school doctor to advise a parent to apply to Mr. So-and-So at such-and-such a hospital than it is for a private practitioner to give a patient his card for the same surgeon's out-patients at the same hospital. The school doctor has merely a professional, semi-scientific interest in the case; neither he nor the surgeon to whom he directs the parent benefits from the case, financially or otherwise—for hospital patients do not, as a rule, remember their surgeons, however much they may express their gratitude to residents and nurses. All that is done in such a contingency is to obtain a definite third or fourth opinion which will to some extent shift the load of responsibility from the shoulders of the school doctor. To him these cases in which operation appears to be urgently desirable are very serious ones. In following up such cases he very often has an opportunity of seeing for himself what mistakes are likely to be made by following a policy of do nothing. Such experience makes him chary of accepting a second opinion, often based on insufficient grounds, which conflicts with his own mature consideration.

